# History, aims and present structure of psychosomatic medicine in Germany

**DOI:** 10.1186/s13030-017-0120-x

**Published:** 2018-01-02

**Authors:** Hans-Christian Deter, Johannes Kruse, Stephan Zipfel

**Affiliations:** 1 Medical Clinic, Psychosomatics, Charité, Hindenburgdamm 30, 12200 Berlin, Germany; 2 Clinic for Psychosomatic Medicine and Psychotherapy, German Association of Psychosomatic Medicine and Psychotherapy (DGPM), University Gießen und Marburg GmbH, Giessen, Germany; 3 Department of Psychosomatic Medicine and Psychotherapy, University Medical Hospital Tuebingen, German College of Psychosomatic Medicine (DKPM), Tuebingen, Germany

**Keywords:** Integrated medicine, Psychotherapy, Research, Teaching, Health care system

## Abstract

Combining its two traditions of integrated psychosomatics in internal medicine and focusing on psychotherapeutic/psychiatric methods in many clinical fields, German psychosomatic medicine has developed well over the last decades. It benefits from its institutional independence and the progressive changes in health care. Clinically, disorder-oriented psychotherapy is a core method integrated with other modes of therapy. As a conceptual base for empirical research, non-reductionist accounts of the interactions of (sick) persons with their environment are most important. Germany has developed a model of Psychosomatic Medicine that has conceptualized and integrated psychotherapeutic methods into clinical practice and realized one way to practice bio-psycho-social medicine in the health care system of the country.

## Background

In this chapter we want to describe the actual situation and the historical development of psychosomatic medicine in Germany. German psychosomatic medicine has different predecessors of current models for bio-psycho-social medicine [1]. It is interesting to understand their historical conditions in a country that has reached an impressive integration in the national health care system [2]. Integrative psychosomatic medicine, which was developed originally in internal medicine, has conceptualized the interactions of mind, body, and social environment.

### History

There has been a tradition of holistic thinking in Germany since the epoch of so-called romantic medicine at the beginning of the 19th century (H. Heinroth, *G.* carus). This was speculative and mystical, and it focused on the unity of body and mind. There was an interest in the “dark side of the soul”, the unconscious and the dreams, which was also reflected by philosophers like A. Schopenhauer, who had worked on the concept of unconsciousness. A strongly scientific medicine developed in the middle of the century, which is tied to the names of W. Griesinger, and R. Virchow, who were also interested in the social aspects of medicine. This discourse about bio-, psycho-, social-medicine never stopped. Despite the rapid development of scientific medicine, a philosophically influenced debate took place at the end of the 19th century. It focused on questions about the relationship between body and mind and on matter and spirit and extended to the beginning of the 20th century [3]. In this time the change from philosophy and psychology (W. Wundt, 1896) to medicine was very close. It is interesting that philosophical schools of phenomenology (E. Husserl), existence philosophy, and hermeneutics (M. Heidegger) had described essentials of the human being as “anthropology”. These philosophers and physicians were familiar with their thoughts. Like the psychiatrist K. Jaspers or the internist V.v. Weizsäcker, they had a strong influence on psychosomatic medicine. This was the climate in which psychosomatic medicine in Germany developed as a basic anthropological orientation in medicine (medicine focused on the individual as a whole and his “being”), as well as an interdisciplinary scientific approach to the study of man and finally a reflection on the applied methods for describing and studying an object and taking into account their limitations [4]. In those days, scientific medicine and knowledge about diseases developed extensively, but L.v. Krehl (1907) was able to state: “We do not treat diseases, but sick people”. Psychosomatic thinking appeared to promote this integration of the human being and his environment.

At the beginning of the last century, German physicians observed an increase in neurotic diseases. War neuroses and functional disorders without organic findings were increasingly seen during World War I (1914–1916). The lack of therapeutic options in the entire field of internal and psychiatric medicine was a reason for developing psychoanalytic oriented psychotherapy and the origins of psychosomatic medicine: Between the two world wars, approaches were made by leading internists V.v. Weizsäcker, G.v. Bergmann, K. Hansen, and coworkers. Moreover, the experience of the national socialist regime and World War II enabled internists like A. Jores to become more familiar with psychosomatic aspects, which they had experienced by themselves or observed in others during that dreadful time. Especially at the internal medicine conference in Wiesbaden in 1949 and by influential internists (T.v. Uexküll, A. Jores et al.), the further institutional development of psychosomatic medicine at universities and in the German health care system was supported. But psychosomatic medicine and its integration in the German academic world and in the area of health care had, compared to other countries, a specific historical, sociological, health policymaking, and medical dimension, which allowed this development and influences it until this day [2]:

#### Historical dimensions

After World War I psychoanalysts established an institute for advanced psychoanalytic training in Berlin and assembled several representatives of the inner circle around S. Freud. The first psychotherapeutic meetings took place under the presidency of C.G. Jung in the 1920íes and brought together all internists, psychiatrists, and therapeutic physicians interested in psychoanalytically oriented psychotherapy. In this time, psychoanalysts and internists involved in psychosomatics (F.Alexander, E. Wittkower) and the founder of GP-discussion-groups (E. Balint) got their training in internal medicine and psychoanalysis in Berlin. Between 1927 and 1930, psychoanalytic clinics were founded by E. Simmel in Tegel and by G. Groddeck in Baden Baden, and a psychosomatic inpatient unit was established in the medical department at the University of Heidelberg by V.v. Weizsäcker. After 1933, the National Socialists (Nazis) fought against psychoanalytic therapy. Most psychoanalysts emigrated and psychoanalytical oriented psychiatrists closed their offices (E. Kretschmer). Only a small group under the protection of a high-ranking administrator (M.H.Göhring) partly identified with the Nazi regime and could continue working in a separate group with a broad range of psychotherapeutic methods (autogenic training by I.H. Schultz, neo psychoanalysis by H. Schultz-Henke). After World War II, two separate psychoanalytic groups (the therapists who stayed on and the emigrants) fought against each other. The emigrants were in close contact with the ideas of the international psychoanalytic society and became official members. They criticized the psychotherapeutic group that remained in Germany, which may have been influenced by Nazi ideology according to their new psychotherapeutic theories and practices. In addition to these groups, psychiatrists (E.Kretschmer) and other clinicians supported the development of psychotherapy in Germany by founding special training sessions (Lindau-psychotherapeutic-week) or scientific journals (Psychotherapy, Psychosomatics and Medical Psychology, 1948). Also, other individuals involved in theory (W. Reich) and the practice of body therapy (E. Gindler, M. Fuchs) have influenced the evolution of German psychosomatic medicine, as well as new psychotherapeutic methods of group therapy, family therapy, and gestalt therapy.

Important to the development of psychosomatic medicine was the physician A. Mitscherlich, who was persecuted and imprisoned by the Nazis and later became (1941) V.v. Weizsäcker’s assistant. He documented and observed the Nuremberg Trials to the extent that they concerned Nazi physicians. When he tried to found an independent psychosomatic institution, he obtained support from the internists, but the head of the psychiatric clinic and other members of the faculty cooperated in the old spirit of the Nazi era and refused a separate department. As a trainee of the Psychoanalytic Institute in London and with funding by the Rockefeller foundation from the USA, he was able to convince German politicians and built his own psychosomatic clinic at the University of Heidelberg.

A similar course took place until 1965 under the protection of the heads of internal medicine departments at the universities of Freiburg, Munich, Hamburg, and Giessen. A number of outpatient and inpatient clinics were set up outside the universities. All of these facilities became nuclei of a development of independent psychosomatic-psychotherapeutic departments outside psychiatric and psychological institutions at many universities (see below).

#### S*ociological*

During the student revolt of 1968, a new generation looked very critically on historical activities of the former generation and was open-minded towards social and psychological problems. This was helpful for a psychosomatic medicine with psychoanalytical theories in competition with faculty members at the universities who were partly identified with Nazi ideas. Psychiatry in Germany after 1945 was essentially directed at organic conditions or psychoses and was less familiar with and interested in psychotherapeutic methods. It had identified itself with the racial laws and ideology of National Socialism, and leading German psychiatrists were involved in Euthanasia and other dreadful activities, but denied this for a long time [5]. They were considered guilty and lost their credibility to the post-war generation.

#### Health policymaking

The development of general prosperity in Germany played a role in this development and the relatively fair distribution of financial resources throughout the German social security system for about 130 years. A number of very active psychotherapeutic associations had a decisive influence on social politics in this time.

#### Medical

In 1967, psychotherapy was included in the German health care system. All patients could get up to 50 sessions of psychotherapy, much more than in other western countries at that time. This was the activity of scientists who could demonstrate the effectiveness of psychotherapeutic treatment [6], but also was a concerted effort by very active groups such as the Physicians society of Psychotherapy, both psychoanalytic societies, the General Practitioner organization, as well as various internists and psychiatrists.

With support of internists (T. v. Uexküll) and psychotherapists (H.E. Richter), psychosomatic medicine was integrated, like in some other Western countries, into medical training at the universities (1970, see below). Experienced and well-known scientists of psychosomatic internal medicine or psychotherapy and psychosomatics became full professors at the universities and built their own independent departments (five as part of medical clinics). To date, 25 departments at 34 medical faculties in Germany have been established, with 22 full professors. The heads of psychosomatic clinics or departments have, as a group, become very influential in Germany and were engaged in the inauguration of both societies.

In 1974, the *German College of Psychosomatic Medicine (DKPM)* was founded by internists and psychotherapists (T.v. Uexküll, P. Hahn, A.E. Meyer, W. Schueffel et al.) in close relation to the thoughts and activities of the International College of Psychosomatic Medicine [7] and the European Conference of Psychosomatic Research (ECPR). The focus of these societies was on psychological aspects of physical symptoms and disorders, and the conferences were very psychotherapeutically oriented with a focus towards psychoanalytic theory. This college (with about 400 members today) was and is an interdisciplinary, integrative scientific professional association with activities in the whole field of medicine for doctors, psychologists, and other professions (e.g. art therapists). It attracted excellent scientists other than those in clinical medicine, psychophysiology (J. Fahrenberg), medical sociology (J. Siegrist), or medical psychology (D. Beckmann), who became members and broadened the scope of this society. In this time close co-operation; a main aim of DKPM; the establishment of many psychosomatic societies of other specialties (e.g. General practitioners, gynecologists, dermatologists, orthopedists) was seen as were psychological societies (medical and clinical psychology) following the concept of (holistic) integrated psychosomatic care in all fields of medicine. In addition to the new development at the universities in the training of all medical students, later it ran and promoted post-graduate research-based training and advancement in all areas of psychosomatic medicine, supported by the Swiss-based Carus Foundation.

A main focus of DKPM scientists was clinical research involving patients of respective hospitals with psychosomatic institutions. Additionally three psychosomatic basic funding fields (SFB’s) of the German Research Council were inaugurated together with other medical disciplines: psychological measurement/psychiatry and psychosomatics (SFB32, Giessen), pulmonary diseases/endocrinology (SFB34, Hamburg) and epidemiology/psychiatry and psychosomatics (SFB116, Mannheim). Later, research exchange formed strong ties to other European, American, and Asian societies involved in the field of psychosomatic medicine. An early exchange of scientists and discussions took place with the American Psychosomatic Society in the 1970íes (H. Weiner, R. Adler, F. Lamprecht). Later, DKPM members were involved in the European C/L study and the following co-operative studies (F. Huyse, U. Malt, T. Herzog, F. Creed). They founded the European Network on Psychosomatic Medicine (2005) and become a member of the International Society of Behavioral Medicine (2008, K. Orth-Gomér, N. Schneiderman). Especially to Japan, there has been a long history of communication with members of the Japanese Society of Internal Psychosomatic Medicine. Our first contacts were with Y. Ikemi, Y. Ago, and H. Suematsu in the 1970ies and 80ies of the last century [8], and now through the ICPM with C. Kubo, Y. Nakai, and M. Murakami. This culminated in the signing of an agreement about Friendship and Cooperation between JSIPM and DKPM in November 2011. Since that time there have been regular exchanges and meetings during national conferences in both countries.

A further and most important step has been with the *introduction of a specialty of Psychotherapeutic Medicine* (this was already installed in the former German Democratic Republic) at the German Physicians Congress in 1992 (P. Janssen. S. O. Hoffmann, H. Schepank). Especially, heads of the departments of psychosomatic medicine committed themselves to establish psychosomatic medicine as an independent specialty in medicine. They were convinced that only a specialization allows intensive training in psychotherapy and psychosomatic medicine. In 1992, the German Society for Psychosomatic Medicine and Psychotherapy was founded. With the unification of this society in 2005 with the Physicians Society of Psychotherapy (AÄGP) - the society for physicians of all specialties trained in psychotherapy, which was founded in 1927 - they changed its name to the *Society of Psychosomatic Medicine and Medical Psychotherapy (DGPM)*. It focused on applying psychotherapeutic and psychosomatic methods at different levels of medicine and in the field of psychosomatic medicine. The society has about 1400 members, mostly practitioners in private practice. The main interest of this society at the beginning was to integrate practical questions of organization, training, and finances of German physicians in the inpatient and outpatient setting. They also wanted influence standards of regular training for this specialty and all other physicians trained in psychotherapy or psychosomatic basic care. This led to co-operation, but later on also to competition with physicians of other specialties, especially with psychiatrists.

In this time and later, the German Society of Psychiatry (DGPPN) was not in favor of the founding of DGPM (and renamed itself later with the adjunct psychosomatic), because at that time in psychiatric organizations, departments, and C/L psychiatry sections there was fruitful co-operation, but also competition in theoretical discussions and practice. The institutional independence of psychosomatic medicine in Germany is largely due to German psychiatry often resisting the integration of psychotherapy as a core method. Some groups gave support for psychotherapeutic methods. Hence, psychosomatic medicine developed independently as an institutional and academic basis for psychotherapy in medicine and later for integrated care models [1]. The independence of the society makes it possible to commit itself to the specific interests of psychosomatic medicine in the German health care system.

#### Common and different scopes of both psychosomatic societies

*Common activities* in the physician organizations and universities in the last 15 years have become very important, including health care, research, physicians/students training, and common conferences. Many professor positions were initiated additionally (e.g. for psychotherapeutic research, psychosomatic genetics, psychosomatic dermatology, etc.), and there was discussion about common and different points of view in both societies to strengthen the power of psychotherapeutic and psychosomatic medicine in Germany.

1. *Basic needs and activities that focus on all physician groups* working in the medical field could be understood by a holistic psychosomatic perspective. The physician’s way of acting on patient satisfaction and on treatment outcome consists of three elements: (1) hands on diagnostic examination (“be-handeln” in German), (2) carry through or organize different additional examinations through colleagues and (3) to communicate and understand patient needs (“be-nennen”- give them a name) and explain diagnostic and therapeutic activities. Physicians in specialties like internal medicine are involved in basic internal care. Interactions between physician and patient are part of their treatment. The special knowledge, experiences, and skills in internal medicine, as well as the communication and behavior of a physician, influence this treatment effect. Thus, societies of internal medicine, psychosomatic internal medicine (like JSPIM), and integrative internal and other specialties (like DKPM) and partly DGPM (which has developed a special training (P. Janssen) are involved in basic care and psychosomatic basic care. Psychotherapists implement additionally verbal, suggestive, or hypnotic therapeutic techniques, which is in depression on a group level not more effective than psychopharmacology. The mean effect size (ES) of psychotherapy compared to no treatment (or treatment as usual) was shown in 15 controlled studies of different diseases to have an ES of .80, but placebo compared to no treatment had an ES of .42, in psycho-diagnostic measurements [9]. Placebo research has taught us that behavior, intake of placebo drugs, or bodily handling have a treatment effect in different diseases. These methods are used by GP’s and other specialties less trained in psychotherapeutic medicine. The goal of “integration of psychosomatic medicine in all clinical fields” is presumably more reachable with a specialty integrated society (e.g. JSIPM) or an integrative society like DKPM with psychosomatic active physicians e.g. GPs, gynecologists, and orthopedists. In-patient settings are common in Germany and show clear advantages in individual stages of psychosomatic or psychological diseases, e.g. anorexia nervosa [10].
2. Competition and cooperation within the medical field: A psychosomatic specialist society like DGPM is compared to others (surgery, internal medicine) in inpatient and outpatient health care and according to influence and power on the same level. Thus, the specialist society may be more effective in collecting institutional and health care advantages. A specialty in psychotherapeutic medicine (e.g. DGPM) will focus on patients, where psychotherapy can be successfully applied. It may focus on diseases, which will be treated effectively by psychotherapeutic and psychosomatic methods. This society can also give support for the development of psychosomatic basic care in the different medical specialties, if these like to cooperate. This is in contrast to psychiatric societies in the medical field, which focus much more on psychopharmacological treatments for severe psychiatric diseases or short psychotherapeutic interventions, in special cases [11].
3. Professional activity: a specialist society like DGPM is focused on physicians as regular members. An interdisciplinary society like DKPM tries to integrate all scientists and clinicians working in the field of medicine: Professionals like psychologists, sociologists, biochemists, physiologists, nurses, and physiotherapists are members and seem to be of the same “rank”.
4. Teaching activities: The teaching of psychosomatic diagnostics and psychotherapeutic methods to students, trainees in their own specialties, and physicians of other specialties is an important task. Both societies were very successful in recent years at the universities, but DGPM worked more in a structural and professional way for the training of physicians and practitioners in an own practice. This training needs cooperation, if the borders between medical fields are unclear and the psychotherapeutic specialist society is interested in working in the fields of specialties, which were involved in the same matter.
5. Research: In its national history Germany developed a holistic psychosomatic and strong psychotherapeutic tradition, which led the way to a psychosomatic/psychotherapeutic specialty. It depends in both societies on experience, wisdom and skills, and the ability to remain or to extend this position and to be successful in competition with other groups working in the field. Due to tradition, clinical activities and competition among psychosomatic scientists in Germany tend to focus on clinically interesting topics relevant to psychotherapeutic activity in some important diseases (see below). DKPM had a broader scope, e.g. in important or other diseases not close to psychotherapeutic activities, in mechanism or public health issues to extend scientific activities in clinical fields, which to date had stimulated only limited interest.
6. International communication and cooperation is needed: a) Many clinicians and scientists from abroad are uncomfortable with the German history and psychosomatic structure in the health care system. If they compare it with their own situation, they are sometimes interested in copying it (but this is not easy, because there are mostly historical and economic differences) or they have completely other ideas and priorities in working in the psychosomatic field in their respective countries, e.g. the International Society of Behavioral Medicine (ISBM) is mainly influenced by psychologists and the Academy of Psychosomatic Medicine (APM), European Association for Consultation/Liaison Psychiatry and Psychosomatics (EACLPP) and its follower European Association of Psychosomatic Medicine (EAPM) by consultation liaison psychiatrists. It takes both information, communication, and discussion of an integrative international based German society like DKPM to communicate freely with all international groups involved in psychosomatic medicine. The combination of psychosomatic medicine and psychotherapy in the DGPM can cooperate also with other psychotherapeutic international organizations. The Society of Psychotherapeutic Research (SPR) and the International Federation of Psychotherapy (IFP) have different scopes than psychosomatic/behavioral international societies. There is an historical development in Germany to combine psychosomatics and psychotherapeutic medicine, so it is a new task to convince international partners involved in psychosomatic medicine or in psychotherapy to cooperate.

Psychosomatic medicine in Germany in the present structures represents a comprehensive field [2] as well as a specialized medical discipline [1]. The two societies, DKPM and DGPM, and their co-operative partners are engaged in activities for patients in health care and for physicians and other care givers in research, training, and national conferences. An English textbook representing psychosomatic medicine in Germany is missing, but a summary of German psychosomatic knowledge has been published [12].

### Patients

Epidemiological studies have shown (depending on the catchment area and “case” definition), that 18.6% to 27.7% of the German population presents psychosomatic, psychiatric, or psychological symptoms that need therapeutic care [13, 14]. Up to 50% of patients in gastrointestinal and other practices suffer under functional or psychic symptoms. 30-40% of inpatients in an internal, neurological, or orthopedic inpatient practice have psychosomatic or psychological symptoms that should be treated [15]. Psychosomatic care is needed on different levels in in- and outpatient settings from regular physicians as well as from specially trained physicians, psychologists, and nurses.

### Psychosomatic professionals in health care

#### Physicians

In 1993, the General Assembly of German Physicians voted for a three-level approach, and thus a differentiated and stepped-care qualification training process, which are as follows:

*Psychosomatic basic care* All clinically practicing medical specialists can enrol in this training. About 35,000 general practitioners and 12,000 gynaecologists have obtained this qualification [16].

#### Additional qualification in psychotherapy

A post-graduate degree in psychotherapy for those already holding a specialization degree (n = about 18,000 [16]).

*Specialization in psychosomatic medicine and psychotherapy* specialists of psychosomatic medicine and psychotherapy (*n* = 5000) [17].

*Psychologists* have special psychotherapeutic training (5 years).

There are several opportunities to train nurses, dietary assistants, physiotherapists, social workers and other care*givers* in a psychosomatic way.

### Psychosomatic care

In Germany, psychosomatic medicine is not a subspecialty of psychiatry, although it has connections in terms of shared models, methods, and overlapping care for patients. It differs in the spectrum of patients (no patients with clear psychosis are treated). Also, based on this differentiation in patient spectrum, for most of the patients the amount of prescribed psycho pharmaceuticals is lower and qualified psychotherapy is higher. At some places, departments of psychosomatic medicine are part of overarching departments for internal medicine. German psychosomatic medicine has focused in recent years on integrated care and therapeutic activities in somatoform/functional disorders, eating disorders, and some somato-psychic disorders in the field of psycho-oncology, psycho-cardiology, pain disorders, and psycho-diabetology. An overlap with other somatic disciplines exists in all somatic specialties, with psychiatry in the fields of depressive, anxiety, and personality disorders.

#### In the *outpatient sector*

There are 3058 specialists in psychosomatic medicine and psychotherapy as well as 3900 specialists in psychiatry and psychotherapy (out of 11,000 psychiatrists), 1972 neurologists (out of 5600 neurologists [18]) and 10,269 medical specialists with an additional psychotherapy certification (out of these, 3493 practice mainly psychotherapy), giving in total of 21.312 medical trained doctors with a certification of psychotherapy and a total of 16,664 psychologists with a specialization in psychotherapy [16]. 64.200 primary care physicians and 64.700 internists are working in Germany [17]. In Germany, only medical doctors and psychologists are allowed to practice psychotherapy for adults.

#### Inpatient therapy

*Consultation/liaison services* for psychosomatic medicine at general and university hospitals exist in some cities with and some without their own inpatient treatment unit. They are separated from psychiatric consultation/liaison services according to patients and applied treatment methods and aims (mainly the bio-psycho-social situation detecting and psychotherapeutic focused) cover 0.5-1.5% of all inpatients in German hospitals. Selected psychiatric C/L services in Germany (emergency focused and pharmacological) cover about 3–3.5% (15). In the European Consultation-Liaison psychiatric service study, 1% of all hospitalized patients were seen [19].

##### Psychosomatic hospitals

Actual data show that there are a total of 223 institutions, covering more than 10,000 inpatient beds, which are responsible for the psychosomatic consultation/liaison service:

- Departments of psychosomatic medicine and psychotherapy as academic institutions at university hospitals. These departments are responsible for patient care (20–70 beds), teaching, and research.
- Hospitals for psychosomatic medicine with a super regional or regional coverage area. Some hospitals include specialized subunits (for example, eating disorders, posttraumatic stress disease, chronic pain).
- Departments of psychosomatic medicine that are part of a psychiatric hospital.

##### Applied methods

Psychosomatic inpatient and day-patient treatment programs are mostly combined multimodal therapy programs, combining psychotherapy in individual and group formats, oriented on treatment guidelines and best practice experiences. Very often, psychodynamic and cognitive-behavioral approaches are both used in a coherent overall framework. They are proven and validated in psychotherapeutic outcome research [10] and applied to different psychological disturbances and psychosomatic diseases. Based on the leading diagnosis, additional therapies e.g. stress-reduction techniques, physiotherapy, body psychotherapy, and creative therapies (art and/or music therapy) are often part of the combined treatment package. Problems in the interpersonal and psychosocial field are in the major focus of family/partner-oriented treatments often supplemented by psychosocial skills training. The mean duration of stay for these programs is 40 days. Health insurance (public or private) covers treatment costs from a budget separate from the one for psychiatry.

##### Quality assurance

The majority of psychosomatic departments and hospitals take part in stringent quality assurance programs in order to monitor their treatment program and to provide transparency to the patients. In addition, in respective regions and states registries were introduced to provide outcome data on basic factors e.g. duration of treatment, leading diagnosis, number of co-morbid disorders [20].

##### Rehabilitation

Aside from the health insurance-covered hospital sector, there is a separate system of rehabilitation centers in Germany that target the clinical areas of cardiology, oncology, neurology, orthopedics, and psychosomatic medicine. Their particular focus is on work-related aspects of disability prevention. Pension insurance covers the costs for these centers. There are 141 centers for psychosomatic medicine with about 16,000 additional inpatient beds [21].

### Research

In a research world increasingly dominated by biological perspectives, bio-psycho-social approaches seem an important activity against reductionism. In the forties, fifties, and sixties of the last century, there was an interest in internal medicine and the psychosomatic aspects of cardiovascular diseases, hypertension, asthma, tuberculosis, in psycho physiology and psychoanalytical description of cases, and also in psychotherapeutic outcome research [6]. Following the nationwide introduction of psychosomatic medical departments in 1970, conducting empirical research did not have priority. Providing proper instruction was more pressing and most of the chairs and professors were psychotherapeutic clinicians and theoreticians rather than empirical researchers. Today, German psychosomatic research plays a more active role:

Clinical studies, especially psychotherapeutic interventions, are the main topics of research in German psychosomatic medicine. Health Research Programs of the German Federal Ministry of Education and Research supported a program of “research groups in psychotherapy”. Few other countries’ research foundations have launched such efforts to facilitate collaboration between research groups in this field. Most studies focus on patients from other specialties with problems that seem to be influenced by psychotherapy (e.g. somatoform disturbances, eating disorders, physical diseases with psychological symptoms). A particular strength of clinical research is the conceptualization, implementation, and evaluation of manualized, disorder-oriented brief psychotherapies tested and demonstrated in different psychosomatic and mental disorders (for example, the ANTOP study in anorexia nervosa [22], the SPIRR-CAD study in depressed patients with coronary heart disease [23] and chronic heart failure [24], the PISO and SPECIAL studies in somatoform disorders [25, 26], as well as the SOPHONET study in social phobia [27], the DAD study in diabetes [28, 29] and RCTs in depression [30, 31]. Psycho-oncology is a fast growing field of research interest in Psychosomatic Medicine, ranging from diagnostic to intervention studies [32, 33]. While many of these therapies are psychodynamic, the scope of treatment development is much broader and also covers CBT-oriented therapy and neuro-psychologically informed treatment approaches. Psychotherapy outcome research has compared different treatments in RCTs. As a second step it is necessary to conduct research to understand which therapeutic factors make the difference. In recent years, the psychotherapy community has worked to modernize web-based health treatments by developing and testing approaches that apply digital media (Interbed, Base, Restart [34, 35]).

#### Co-working in guidelines

Developing and coordinating evidence-based national guidelines on topics relevant to psychosomatic medicine, such as functional/somatoform or eating disorders, has been an important undertaking in recent years [25, 36, 37].

Examples for public health and healthcare research.

#### Public health

Researchers of psychosomatic medicine have participated in several population-based cohorts in Germany (for example, the Gutenberg Health Study in Mainz [38], the KORA study in Augsburg, and the ESTHER study in Heidelberg [39]). The studies provide epidemiological data on the interaction of different psychological, social, immunological, and other risk factors in the etiology and course of diabetes and cardiovascular and other chronic diseases.

#### Psychosomatic-based healthcare research

- *regional networks of patients with eating and somatoform disorders:* A trans-sectorial networking initiative in the city of Hamburg brought together more than 60 partners from the government and the research, healthcare, and health industries (“Psychenet” [40]). In cooperation with other academic partners, the local university’s Department of Psychosomatic Medicine coordinates and evaluates screening, networking, and therapeutic interventions [41].
- *psychodiagnostic measurement in psycho-oncology:* Typical topics include developing screening instruments [30], assessing the need for psycho-oncological support for patients and caregivers [42], and evaluating naturalistic interventions [43].

#### Examples for basic science

There is a tradition of basic science-related research [44], especially in the areas of mechanism-oriented, psycho-physiological [45], life event, and stress-related research [46]. In the last decade though, there has been an increase in the number of institutions with expertise in the field of neuroscience-related research paradigms including neuro-immunology, neuro-cognitive, and neuro-imaging approaches [47], which cooperate in a psychobiological perspective with other professional groups in the individual research field.

Psychosomatic medicine research also plays a role in national and international collaborative research networks (on nutrition medicine and eating disorders, placebo and nocebo effects, and on the neural control of intestinal functions). Interest and expertise in animal-based or wet lab-based research is relatively recent.

#### Conceptual work

Neuroscientists, psychologists, psychiatrists, and philosophers in cooperation with psychosomatic scientists worked on adapting bio-psycho-social concepts to modern medicine in two interdisciplinary European Union Marie Curie Training Networks (“DISCOS” and “TESIS” [48]. New own studies and research questions are discussed also between neuroscience and philosophy in the Mind and Brain DFG founded graduate college at the Charité in Berlin (www.mind-and-brain.de).

### Training

#### Academic teaching

In 1970, psychosomatic medicine became an obligatory subject in the medical school curriculum at the 37 official medical schools in Germany. Building on this advancement, the majority of the medical schools (*N* = 26) then decided to establish departments of psychosomatic medicine. These departments’ curricula cover the topic of psychosomatic medicine - its disorders, diagnostic techniques, and treatment methods, but department members have actively created and implemented communication skills training as part of the medical curriculum. The departments have also been involved in the development, implementation, and evaluation of state-of-the-art teaching formats and continue to play an active role in the international medical education community [49].

#### Professional training

##### Psychosomatic basic care

All physicians with a specialty can take part in an 80-h training, which includes theoretical and clinical instruction, communication training with psychosomatic patients, and reflection on the doctor-patient relationship.

##### Additional qualification in psychotherapy

A post-graduate degree in psychotherapy for those already holding a specialization degree. The training is extra-occupational and conveys basic psychotherapeutic skills (120 h of supervised psychotherapy, 120 h of theoretical input and personal therapy).

*Specialization in psychosomatic medicine and psychotherapy* (5-years, including 1 year each of internal medicine and psychiatry). This training comprises 1500 h of supervised psychotherapy, 240 h of theoretical input, and personal therapy in both individual and group settings. (for more details see [50]).

### Organization of National Congresses

Together, the DKPM and DGPM have organized since 2007 a common conference on psychosomatic medicine and psychotherapy at the end of March each year. This is an umbrella meeting for all scientists, professionals, and care providers active in psychosomatic medicine in Germany, but also other countries like Austria and Switzerland. All active groups and societies in this field are invited to participate and to present their research and experiences. A part of the conference is reserved for training in psychosomatic medicine and psychotherapy for physicians, psychologists, and students. In recent years, on average 1100 participants and around 450 papers were presented in symposia or poster sessions including an English/international track. This conference is not sponsored by the pharmaceutical sector.

## Conclusion

Psychosomatic Medicine in Germany now represents a comprehensive field [51] as well as a specialized medical discipline [1]. It has a larger institutional basis than in any other country and is not related to consultation liaison psychiatry [52]. The progress in care, research, and training could serve as an example for international discussions. International exchange may stimulate a progression to implement in respective countries different aspects of psychosomatic medicine in care, research, and training of students, physicians and other professionals involved in psychosomatic medicine [53].
